# Impact of effective prevention and management of febrile neutropenia

**DOI:** 10.1038/sj.bjc.6605273

**Published:** 2009-09-15

**Authors:** D Krell, A L Jones

**Affiliations:** 1 Department of Academic Oncology, Royal Free Hospital, Pond Street, London NW3 2QG, UK; 2 Department of Academic Oncology, University College Hospital, 250 Euston Road, London NW1 2PG, UK

**Keywords:** cost, febrile neutropenia, prevention, treatment, protocols

## Abstract

Chemotherapy-induced febrile neutropenia is costly in both financial and human terms. The associated costs can be reduced substantially through the development and implementation of national policies and locally agreed protocols for the prevention and management of febrile neutropenia. Patients, the NHS, healthcare professionals and the broader community all stand to benefit from a commitment to effective management of this common and predictable side effect of some chemotherapy regimens for early-stage breast cancer.

Febrile neutropenia (FN) is a serious complication of chemotherapy for early-stage breast cancer, with significant morbidity and mortality, and important implications for patients and healthcare resources.

It is estimated that 95% of women diagnosed with breast cancer in the UK have early-stage disease (43 000 women/year) (CRUK, 2009), 13 000 (30%) of whom will be node-positive (Verschraegen *et al*, 2005). Around 9000 women per year with node-positive early-stage breast cancer receive chemotherapy (CRUK, 2009). On the basis of the regimens used and their reported FN rates, the incidence of FN among women receiving chemotherapy for node-positive early-stage breast cancer is estimated at around 16% (Poole *et al*, 2006; Roché *et al*, 2006; Ali *et al*, 2008; Head *et al*, 2008; Scaife *et al*, 2008; Zaman *et al*, 2008). Therefore, it is conservatively estimated that more than 1000 women each year receiving chemotherapy for node-positive breast cancer will have an episode of FN (Poole *et al*, 2006; Roché *et al*, 2006; Ali *et al*, 2008; Head *et al*, 2008; Scaife *et al*, 2008; Zaman *et al*, 2008).

FN requires hospitalisation and treatment with intravenous antibiotics, and has a negative impact on patients’ quality of life (Moore and Crom, 2006). Furthermore, the development of FN may lead to a decision to reduce or delay the patient's subsequent chemotherapy dose, which can undermine treatment outcomes, including overall survival, particularly in the adjuvant setting (Bonadonna *et al*, 1995; Lyman *et al*, 2005; Chirivella *et al*, 2006).

The effective management of FN embraces both prevention of the condition with prophylactic measures, such as the use of granulocyte colony-stimulating factors (G-CSF) and/or antibiotics, and the appropriate management of febrile neutropenic events, notably neutropenic sepsis.

Other articles in this supplement look in detail at FN prevention and management (Kelly and Wheatley, 2009; Cullen and Baijal, 2009). In this article, we consider the impact of prophylactic and management interventions on patients, the NHS, healthcare professionals and the broader community.

## Impact of FN prophylaxis

**Figure 1:**
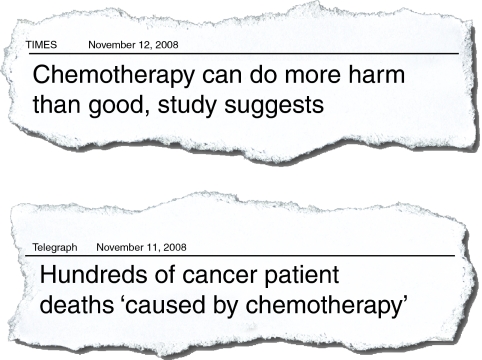
National headlines reflect how public confidence in cancer services is undermined.

If a patient develops FN, the direct and indirect costs to the individual, the NHS and the economy are substantial. The costs derive from a range of factors, including hospitalisation for treatment of FN, significant morbidity and mortality, financial losses for patients and their families/carers and reduced health-related quality of life (Moore and Crom, 2006; Gridelli *et al*, 2007). These costs also undermine public confidence in cancer services (Figure 1).

Furthermore, the loss of productivity associated with hospitalisation and morbidity has a detrimental impact on the economy.

**Figure 2:**
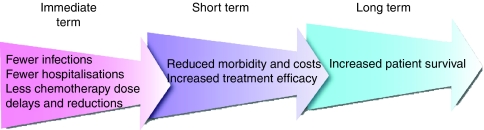
Short-term and long-term effects of FN prevention.

By preventing FN through the use of appropriate prophylactic measures, some of these costs may be avoided (Figure 2), leading to improved quality of life and treatment efficacy for patients, reduced healthcare costs and greater predictability of care needs (Kuderer *et al*, 2006).

As discussed by Trueman (2009) in this supplement, analysis of the cost effectiveness of prophylaxis with G-CSF is fraught with difficulty, because of a lack of consistency across clinical trials and because of the problems faced when transferring pharmacoeconomic considerations from one healthcare system to another. However, various economic models suggest that primary prophylaxis with G-CSF may be cost-effective when the risk of FN exceeds specific thresholds, for example, 20% (Lyman *et al*, 1998), 16% (Eldar-Lissai *et al*, 2008) or 18% (Dale *et al*, 2006).

## Impact of management of FN events

Inadequacies in the management of FN in the UK, and several key organisational and clinical failures (Table 1), were highlighted recently by the National Confidential Enquiry into Patient Outcome and Death (NCEPOD, 2008). Such inadequacies have a negative impact on patients, and on the perception of cancer care by the broader NHS and the public. This perception leads, in turn, to reduced confidence in the NHS, which, coupled with the added stress caused by potentially avoidable additional hospital visits and extended hospitalisation, may affect patients’ willingness to undergo further treatment (Fortner *et al*, 2002).

In response to the findings of NCEPOD and a report from the National Cancer Peer Review Network (NCPRN, 2008), the National Chemotherapy Advisory Group has issued recommendations for improving the management of FN (NCAG, 2009). The advice is aimed not only at chemotherapy providers but also at any hospital with acute facilities to which patients with possible chemotherapy side effects may present. The implementation of the recommendations is expected to benefit patients, the NHS and healthcare professionals.

### Benefits for patients

Effective management of FN events may have a significant impact on patients’ quality of life, morbidity, mortality, long-term survival and finances.

The development of FN has been shown to correlate with lower quality-of-life scores (Okon *et al*, 2002) and an increase in the incidence and severity of chemotherapy-related side effects such as mucositis, abdominal pain and diarrhoea, anorexia and fatigue (Glaspy *et al*, 2001).

The mortality rates associated with FN range from 2 to 21% (Smith *et al*, 2006; Herbst *et al*, 2008) – the higher rates are often seen in patients with comorbidities, which may be age-related, or in patients with poor performance status, including those with advanced cancer and undergoing palliative chemotherapy (Lyman *et al*, 2005).

FN often results in chemotherapy dose reductions and dose delays (Leonard *et al*, 2003), and the resulting reduction in chemotherapy dose intensity can have a significant negative impact on clinical outcome, notably survival (Bonadonna *et al*, 1995; Chirivella *et al*, 2006). Indeed, patients who receive less than 65% of their planned dose have been shown to have survival rates similar to those who receive no chemotherapy at all (Bonadonna *et al*, 1995). Such dose reductions are common in the absence of clear local policy on primary prevention of neutropenia in patients undergoing treatment with curative intent. However, dose reductions (and lower starting doses) may be advisable in certain high-risk patients, including those with comorbidities or poor performance status and those undergoing palliative chemotherapy, who are also susceptible to the non-haematological toxicities associated with chemotherapy (Lyman *et al*, 2005; NCEPOD, 2008).

FN disrupts normal life activities such as childcare and employment (Moore and Crom, 2006), and thus has financial and social implications for patients and their families.

### Benefits for the NHS

FN imposes a significant burden on NHS finances and resources – a single episode is estimated to cost the NHS £3582 (Holmes *et al*, 2004). The major economic impact is related to hospitalisation; the average length of hospital stay is 6–8 days (Kuderer *et al*, 2006). Hospitalisation puts patients at risk of developing further complications, such as hospital-acquired infections and thromboembolic events, which add to the overall cost of FN.

Inadequate admission pathways and a lack of management protocols lead to inappropriate placement of patients, inefficient and inappropriate use of healthcare resources, treatment delays and prolonged hospitalisation (NCEPOD, 2008). Implementation of network policies, and locally agreed hospital admission pathways, together with the availability of clear management protocols, should lead to patients with FN being admitted efficiently, under the care of the appropriate healthcare professional and receiving the appropriate treatment in a timely manner (NCEPOD, 2008). Such practices will reduce healthcare costs, encourage appropriate and efficient use of NHS resources and reduce the costs associated with prolonged stay, morbidity and mortality.

### Benefits for healthcare professionals

As a result of locally agreed and implemented policies for the management of FN, healthcare professionals will become more confident in their ability to manage patients with the condition, and the public's confidence in healthcare services will be enhanced. Achieving this goal will require education of all healthcare professionals, including those not directly involved in frontline cancer care, both at a national and local level (NCEPOD, 2008). It will also require a regular, systematic audit of the complications of chemotherapy, with review at a local and network level forming part of the appraisal and education of healthcare professionals (NCEPOD, 2008).

## Impact of change

Several groups have conducted audits in patients receiving chemotherapy, looking at the rates of FN, dose delays and dose reductions, and the effects of adding prophylactic G-CSF and/or antibiotics (Ali *et al*, 2008; Head *et al*, 2008; Scaife *et al*, 2008). The results of three audits of patients receiving FEC-T (fluorouracil, epirubicin and cyclophosphamide followed by docetaxel) or TAC (docetaxel, doxorubicin and cyclophosphamide) were presented at the 2008 National Cancer Research Institute Cancer Conference, which took place in Birmingham. The findings are summarised in Tables 2,3,4. All three groups plan to re-audit their practice following the introduction of primary prophylaxis with G-CSF with or without antibiotics in the same groups of patients. The findings of these re-audits may prove useful in guiding the management of FN in the future.

## Conclusion

FN is a significant complication of chemotherapy treatment from the point of view of patients, healthcare professionals and the NHS. Reports of serious inadequacies in the management of FN in the UK (NCEPOD, 2008; NCPRN, 2008) have led to recommendations (NCAG, 2009) for robust systems to be put in place to admit and manage patients with FN. The implementation and audit of such systems will have an impact not only on individual patients but also on healthcare professionals and the wider NHS.

## Figures and Tables

**Table 1:** Failures in the management of patients admitted with neutropenic sepsis (NCEPOD, 2008)

**Organisational failures**	**Clinical failures**
Lack of treatment policy in emergency departments	Delayed admission
Clinician unaware of treatment policy	Failure of junior doctors to make the diagnosis
Patient managed in an inappropriate care setting	Lack of awareness that patients without a fever may still have FN
Only occasional oncology visit to cancer unit in a district general hospital	Lack of early assessment by senior staff Delayed resuscitation Delayed prescription and administration of antibiotics Failure to adhere to local antibiotics policy Delayed transfer to intensive care

**Table 2:** Rates of FN associated with the use of adjuvant FEC-T chemotherapy in high-risk node-positive patients with early breast cancer: a UK perspective (Head *et al*, 2008)

**Data audited**	**Patients audited**	**Audit findings**	**Recommendation**	**Plan to re-audit**
FN rate	137	25%	Primary G-CSF should be used throughout treatment with FEC-T	Following introduction of primary prophylaxis with G-CSF
Dose delays		25/137 (18.2%)		
Dose reductions		27/137 (19.7%)		
Use of primary G-CSF		30 patients		
FN rate after primary G-CSF		2/30 (8.5%)		
Use of secondary G-CSF		25%		
FN rate after secondary G-CSF		0		

**Table 3:** Experience of FN and secondary G-CSF prophylaxis during FEC-T chemotherapy in the Merseyside and Cheshire Cancer Network (Ali *et al*, 2008)

**Data audited**	**Patients audited**	**Audit findings**	**Recommendation**	**Plan to re-audit**
FN rate	123	33/123 (27%)	Primary or secondary prophylaxis may be indicated with FEC-T	Following the introduction of primary prophylaxis with antibiotics
Cycles complicated by FN		39/728 (5.36%)		
Use of primary G-CSF		3 patients		
FN after primary G-CSF		2 patients		
Use of primary prophylactic antibiotics		3 patients		
FN after primary prophylactic antibiotics		0 patients		
Use of secondary G-CSF		24 patients		
Episodes of FN after secondary G-CSF		2/24 (8%)		

**Table 4:** FN in patients receiving TAC chemotherapy for breast cancer (Scaife *et al*, 2008)

**Data audited**	**Patients audited**	**Audit findings**	**Recommendation**	**Plan to re-audit**
One or more episodes of FN	49	16/49 (33%)	Quinolone antibiotics should be commenced 5 days post-chemotherapy	Re-audit following the introduction of primary prophylaxis with both G-CSF and antibiotics
Episodes of FN in cycle 1		8/16 (50%)		
Dose delay or reduction		13/16 (81%)		
Death due to sepsis		1 patient		
Median duration of inpatient stay for FN		4 days		
